# Supplementary figures and images for: Decreased Protein Kinase C-β Type II Associated with the Prominent Endotoxin Exhaustion in the Macrophage of FcGRIIb−/− Lupus Prone Mice is Revealed by Phosphoproteomic Analysis
Source: Int J Mol Sci. 2019 Mar 18;20(6):1354. doi: 10.3390/ijms20061354 (PMC6472018; doi:10.3390/ijms20061354)

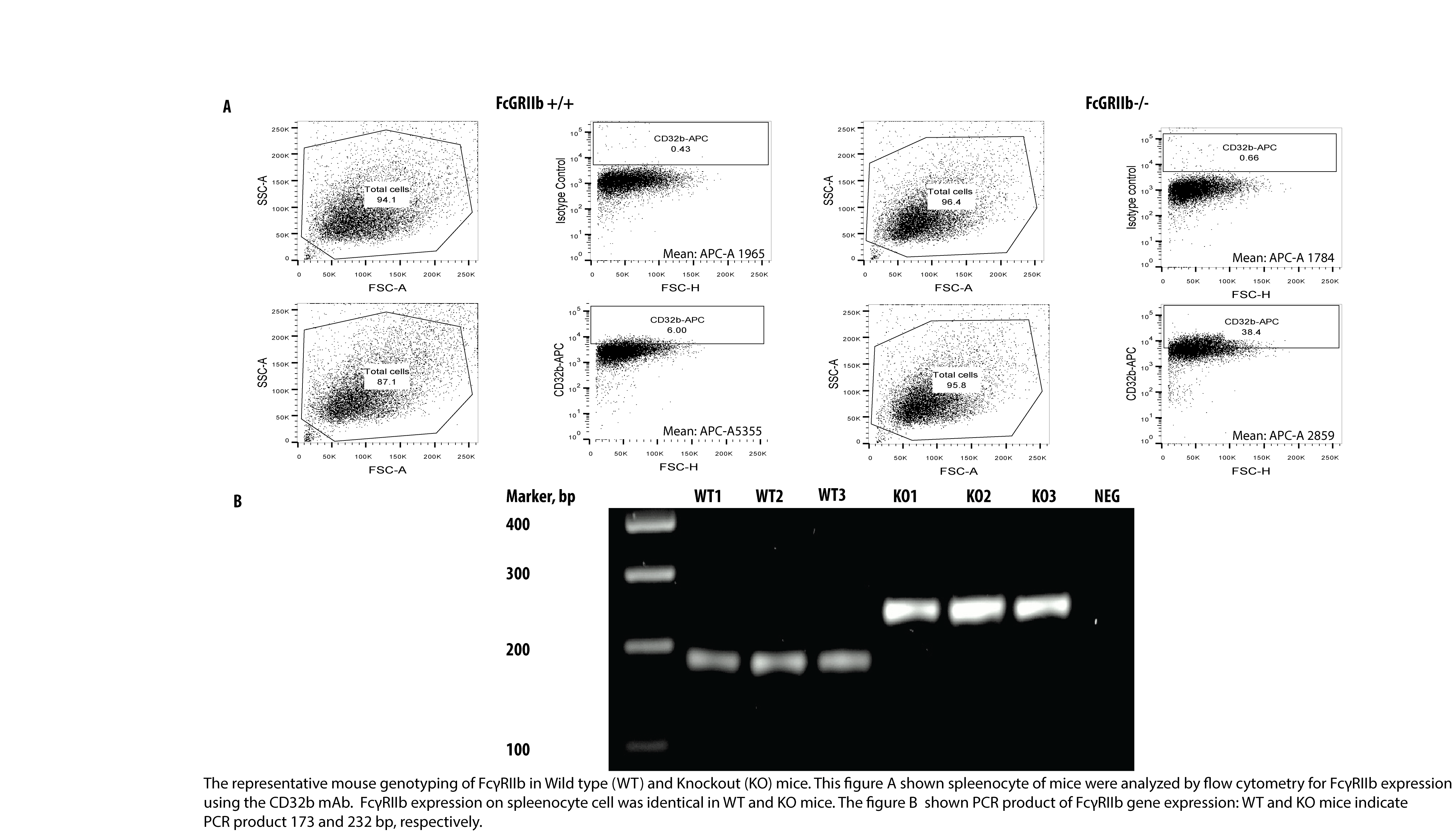

Supplement: Supplementary file 1 [file ijms-20-01354-s001.zip › Figure S1.tif]
